# Lessons from Neurotrauma Treatment Simulation Center (NTSC) – The neurologist's perspective

**DOI:** 10.25122/jml-2022-1016

**Published:** 2022-07

**Authors:** Iulia Vadan, Victor Dabala, Dafin Fior Muresanu

**Affiliations:** 1. Department of Neuroscience, Iuliu Hatieganu University of Medicine and Pharmacy, Cluj-Napoca, Romania; 2. RoNeuro Institute for Neurological Research and Diagnostic, Cluj-Napoca, Romania

## INTRODUCTION

Neurologists worldwide consider traumatic brain injury (TBI) an important healthcare, medical, and societal problem, being a major cause of morbidity and mortality in young people. In high-income countries, the TBI incidence is rising in people aged 65 years or older, representing an important cause of long-term disabilities among survivors and dramatically affecting their daily life and social activities and the life of their family and friends [1]. In Europe, where there are differences between countries, especially based on their income, research shows that the incidence of TBI is about 280.5/100,000.

### NTSC – Expanding the multidimensional approach

Neurotrauma, mainly represented by TBI, is one of the most prevalent conditions diagnosed in emergency neurology, impacting a critical number of patients annually. Significant efforts towards promoting research standards have been made in recent years, with the Academy for Multidisciplinary Neurotraumatology (AMN), set up in 2004, being among the main drivers behind the success of these efforts. The purposes of this academic society reside in the continuous development of scientific and clinical aspects of neurotraumatology through a multidimensional approach.

The first edition of the Neurotrauma Simulation Center (NTSC), held between the 16^th^ and the 20^th^ of May 2022, represented an ambitious project where delegations from different countries (Egypt, Mexico, the Philippines, Poland, Romania, Slovakia) gathered doctors with different specialties involved in the pathway of TBI patients, trying to assess the current global issues that impact neurotraumatological care. One main point agreed upon by the participants was the dire need for a multidisciplinary approach to target the best treatment and outcome for patients.

The NTSC experience exposed various possibilities for improvement that could be further implemented in countries where TBI management can benefit from the guidance of high-level know-how showcased in Vienna.

### The creation of a unique TBI – related program

Changing the approach in neurotrauma implies several aspects mainly based on shifting the paradigm from short-term focus to long-term care for the neurotrauma patient.

The 2022 NTSC edition focused on sharing personal experiences, practical approaches, and countries' TBI *status quo* while simultaneously acknowledging the recovery chain of TBI in the Austrian healthcare system. Consequently, four medical centers (Vienna General Hospital, The Bad Pirawarth Therapeutic Rehabilitation Centre, the Floridsdorf Klinik, and Landesklinikum Wiener Neustadt) were selected for the on-site activities.

The entire program was based on the dichotomy between theoretical and practical approaches, the first one bringing insight into the importance of human resources in the entire recovery chain of the patient, and the latter allowing all the specialists the opportunity to exercise their medical skills. The novelty of the recovery chain resides not only in the fast-response to a neurotrauma case, as the accent is placed on increased communication and coordination among all specialists (neurologist, neurosurgeon, anesthesiologist, traumatologist, rehabilitation specialist) but also on ensuring an appropriate and comprehensive long-term follow-up for the patient. All of these were practically illustrated in the simulation procedures at Floridsdorf Klink on the last day of the program. Here, all specialists took part in complex exercises depicting simulated neurotrauma cases, when all their clinical, interpersonal, intrapersonal, leadership, and teamwork skills were tested. Team formation and team management were also skills exercised during the rotation exercises at Vienna General Hospital, which included the Emergency Department, ShockRooms, Intensive Care Unit (ICU), and Neurosurgery ward. Here, the faculty representatives offered insight on setting up a TBI Team. The rotation exercises were completed with different simulations, from the radiology diagnostic in a TBI case to the operating rooms, as well as presentations of the ICU equipment and the intracranial pressure (ICP) monitoring of the patients. As the rehabilitation process has become a very complex and integrative element of the long-term follow-up, the Therapeutic Rehab Centre in Bad Pirawarth offered insight into these procedures in the Austrian healthcare system. As such, hands-on activities were conducted on the fourth day of the program, ranging from occupational therapy and physical therapy, swallowing assessment, FEES (fiberoptic endoscopic evaluation of swallowing), orthoptic treatment and evaluation, and neuropsychological discussions conducted with a post-TBI patient.

Important lessons of the program were highlighted during the country implementation plans regarding TBI treatment, presented within the ending session of the NTSC. As the lack of a national/international TBI registry was the main limitation evoked by the participants, special attention was given to PRESENT (Patient REgistry – Short Essential NeuroTrauma), a registry set up to collect multidisciplinary, multidimensional, and longitudinal data from all care levels of TBI and to serve as a go-to hub for basic TBI data collection in all countries, both in those with low access to data surveillance mechanisms, as well as in more advanced healthcare systems. The multimodal approach of the registry involving all specialties ensures the measurement of the true standard of care and helps generate performance improvement indicators by following the patient's pathway from early acute care to post-acute care and neurorehabilitation services. It is worth mentioning that the entire week highlighted the common goal of such a program, namely the improvement of the patient's treatment, outcome, and quality of life. To achieve this, various topics on neurotraumatology were approached, completed with lively discussions where participants shared their perspectives.

From a practical point of view, a good example of implementation is definitely represented by the development of an air rescue system for specific countries, especially those with a mountain range across their territory (*e.g*., Poland, Romania, or Slovakia) in contrast to other territories (*e.g*., Egypt), where most landforms are not represented by elevated terrain. Furthermore, in countries where TBI management is centralized (multiple departments housed in a single structure), dividing critical care and neurorehabilitation processes by decentralization could ease specific aspects of the burden of neurotrauma.

## CONCLUSIONS

From the neurologist's perspective, TBI is often encountered as one of the most frequent pathologies in clinical practice. The extraordinary Neurotrauma Treatment Simulation Center (NTSC) program and the chance to debate and discuss with specialists from different countries regarding the treatment, procedures, and rehabilitation process is of great benefit, not only for specialists but for patients as well.

The efforts that different academic societies, including the Academy of Multidisciplinary Neurotraumatology (AMN), have put forward in the last years to help doctors and improve the patient outcome and rehabilitation must be recognized and applauded, as only through constant efforts performance improvement indicators could be developed, and the TBI treatment could be standardized at a global level.
